# Clinical Validation of an Artificial Intelligence–Based Tool for Automatic Estimation of Left Ventricular Ejection Fraction and Strain in Echocardiography: Protocol for a Two-Phase Prospective Cohort Study

**DOI:** 10.2196/44650

**Published:** 2023-03-13

**Authors:** Stelios Hadjidimitriou, Efstathios Pagourelias, Georgios Apostolidis, Ioannis Dimaridis, Vasileios Charisis, Constantinos Bakogiannis, Leontios Hadjileontiadis, Vassilios Vassilikos

**Affiliations:** 1 Department of Electrical and Computer Engineering Aristotle University of Thessaloniki Thessaloniki Greece; 2 Third Cardiology Department Hippokrateion Hospital, Medical School Aristotle University of Thessaloniki Thessaloniki Greece; 3 Department of Biomedical Engineering Khalifa University Abu Dhabi United Arab Emirates; 4 Healthcare Engineering Innovation Center Khalifa University Abu Dhabi United Arab Emirates

**Keywords:** artificial intelligence, clinical validation, computer-aided diagnosis, echocardiography, ejection fraction, global longitudinal strain, left ventricle, prospective cohort design, ultrasound

## Abstract

**Background:**

Echocardiography (ECHO) is a type of ultrasonographic procedure for examining the cardiac function and morphology, with functional parameters of the left ventricle (LV), such as the ejection fraction (EF) and global longitudinal strain (GLS), being important indicators. Estimation of LV-EF and LV-GLS is performed either manually or semiautomatically by cardiologists and requires a nonnegligible amount of time, while estimation accuracy depends on scan quality and the clinician’s experience in ECHO, leading to considerable measurement variability.

**Objective:**

The aim of this study is to externally validate the clinical performance of a trained artificial intelligence (AI)–based tool that automatically estimates LV-EF and LV-GLS from transthoracic ECHO scans and to produce preliminary evidence regarding its utility.

**Methods:**

This is a prospective cohort study conducted in 2 phases. ECHO scans will be collected from 120 participants referred for ECHO examination based on routine clinical practice in the Hippokration General Hospital, Thessaloniki, Greece. During the first phase, 60 scans will be processed by 15 cardiologists of different experience levels and the AI-based tool to determine whether the latter is noninferior in LV-EF and LV-GLS estimation accuracy (primary outcomes) compared to cardiologists. Secondary outcomes include the time required for estimation and Bland-Altman plots and intraclass correlation coefficients to assess measurement reliability for both the AI and cardiologists. In the second phase, the rest of the scans will be examined by the same cardiologists with and without the AI-based tool to primarily evaluate whether the combination of the cardiologist and the tool is superior in terms of correctness of LV function diagnosis (normal or abnormal) to the cardiologist’s routine examination practice, accounting for the cardiologist’s level of ECHO experience. Secondary outcomes include time to diagnosis and the system usability scale score. Reference LV-EF and LV-GLS measurements and LV function diagnoses will be provided by a panel of 3 expert cardiologists.

**Results:**

Recruitment started in September 2022, and data collection is ongoing. The results of the first phase are expected to be available by summer 2023, while the study will conclude in May 2024, with the end of the second phase.

**Conclusions:**

This study will provide external evidence regarding the clinical performance and utility of the AI-based tool based on prospectively collected ECHO scans in the routine clinical setting, thus reflecting real-world clinical scenarios. The study protocol may be useful to investigators conducting similar research.

**International Registered Report Identifier (IRRID):**

DERR1-10.2196/44650

## Introduction

### Background

Echocardiography (ECHO) is a type of ultrasonographic scan used for examining the cardiac function and morphology [1]. Due to the practicality of image acquisition, relatively low cost, and little-to-no-risks for the patient, ECHO remains the most widely used cardiac imaging examination [2]. Of pivotal importance for patient evaluation is the assessment of the left ventricular (LV) systolic function [3], with recommended evaluation parameters being the ejection fraction (EF) and global longitudinal strain (GLS) [4]. EF is the percentage of blood ejected from the ventricle in each contraction, measured as the ratio of stroke volume (difference between end-diastolic and end-systolic volume) over the end-diastolic volume. GLS describes the myocardial deformation across the ventricular longitudinal axis, measured as the relative length change of the ventricular myocardium between end-diastole and end-systole.

Estimation of LV-EF and LV-GLS from ECHO scans is performed by cardiologists either manually or semiautomatically using a dedicated software [5]. One or more cardiac cycles and apical views of the LV can be used in the estimation of both parameters. Different apical views (typically 4-chamber, 2-chamber, and apical long axis views) are obtained by different placements of the ultrasound probe relative to the heart apex [1]. The estimation process is usually laborious and time-consuming, especially when considering medical emergencies, while accuracy depends on the cardiologist’s experience in ECHO and the quality of scans [6], leading to considerable measurement variability [4,7]. Improving measurement precision and reliability is necessary, as it has been shown that EF and GLS values and their impairments are closely connected to patients’ prognosis and course [8,9].

Deep learning [10], the most recent advancement in artificial intelligence (AI), has revolutionized computer vision over the past decade. Deep neural networks can be trained on annotated images without the need for feature engineering and perform fully automated image analysis. Inevitably, deep learning–based systems have been used in medical imaging, with a recent surge in applications for cardiovascular imaging [11]. Such advanced AI and associated software could support cardiologists of various levels of expertise in LV function assessment with the automatic and more accurate estimation of LV-EF and LV-GLS, and at the same time reduce the time of ECHO examination and the variability of measurements. In conjunction with point-of-care ultrasonography, such AI-based assistance may significantly improve relevant case management in demanding health care settings, such as emergency departments.

### Objectives

This study aims at evaluating the clinical performance and producing preliminary evidence regarding the utility of a trained AI-based tool that automatically estimates LV-EF and LV-GLS from transthoracic ECHO scans. Two study phases are planned. The specific primary and secondary objectives of each study phase are listed in Textbox 1.

**Primary and secondary objectives of the 2 phases of the study.**

**Phase 1**

- Primary objective
  Determine if the automatic estimation of left ventricular ejection fraction and left ventricular global longitudinal strain from echocardiography scans by the artificial intelligence–based tool is noninferior in terms of accuracy to semimanual estimations by cardiologists of different experience levels.
- Secondary objective
  Evaluate the time required for left ventricular ejection fraction and left ventricular global longitudinal strain measurement from echocardiography scans by the artificial intelligence–based tool and by semimanual estimations of cardiologists of different experience levels.

**Phase 2**

- Primary objective
  Determine if the combination of the cardiologist and the artificial intelligence–based tool is superior to the routine examination practice of the cardiologist in terms of correct diagnosis of left ventricular function (normal or abnormal) based on examination of echocardiography scans, accounting for the cardiologist’s level of experience.
- Secondary objective
  Evaluate the time required for diagnosis with and without the assistance of the artificial intelligence–based tool.
  Evaluate the usability of the artificial intelligence–based tool based on cardiologists’ feedback after use.

## Methods

### Study Design

**Figure 1:**
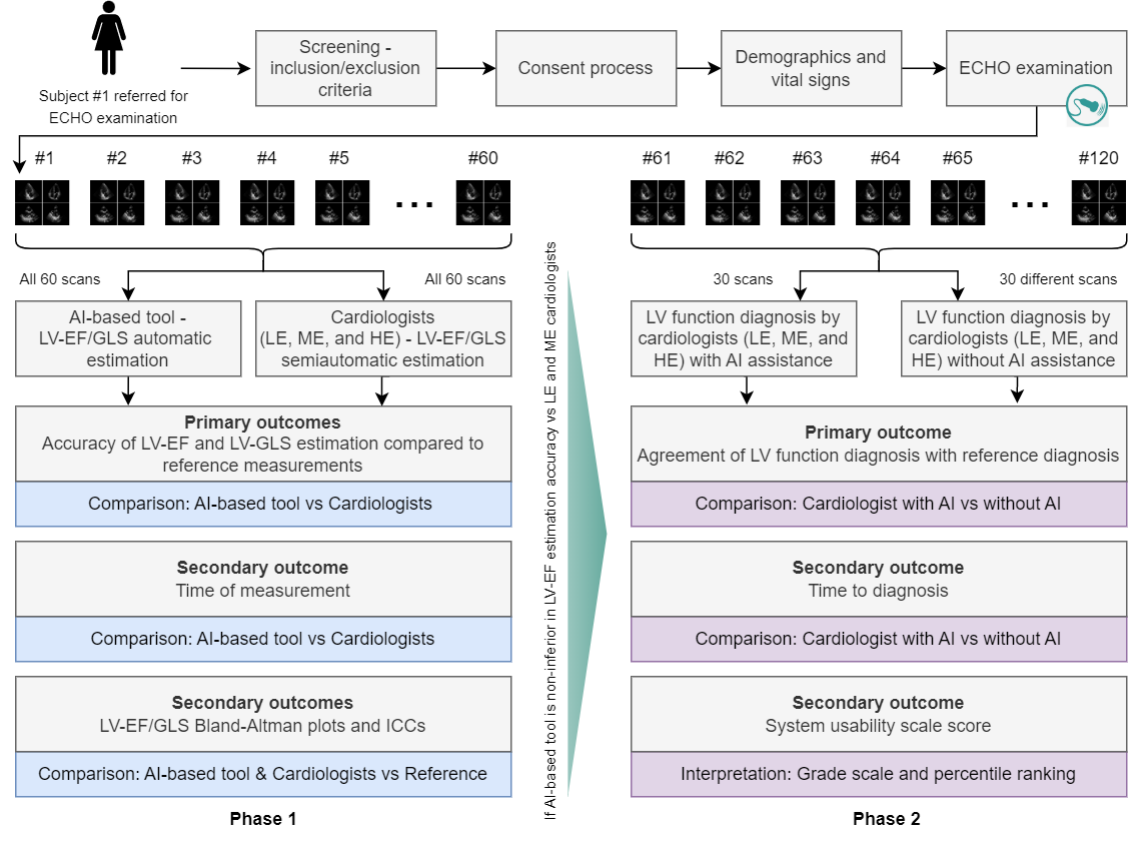
Study overview. After enrollment in the study, an ECHO scan will be acquired from each participant. In total, 120 scans are planned to be collected, with 60 scans used in the first phase of the study evaluating the performance of the AI-based tool in LV-EF and LV-GLS estimation compared to the semimanual estimation by cardiologists of different experience levels and the rest in the second phase evaluating the utility of the AI-based tool in LV function diagnosis by cardiologists of different experience levels. AI: artificial intelligence; ECHO: echocardiography; EF: ejection fraction; GLS: global longitudinal strain; HE: high experience; ICC: intraclass correlation coefficient; LE: low experience; LV: left ventricle; ME: medium experience.

This is a prospective cohort study conducted in 2 phases, involving multireader, multicase diagnostic measurement, and diagnostic performance evaluation. Data (ECHO scans) collection is common between the 2 phases. The specific objectives of each study phase were detailed in Textbox 1. In both phases, reference measurements and reference diagnoses will be provided by a panel of 3 highly experienced cardiologists, via a collective intelligence process. We will proceed with the second phase if and when the AI-based tool is proven to be noninferior in terms of (at least) LV-EF estimation compared to (at least) low- and medium-experienced cardiologists in the first phase. Figure 1 provides an overview of the study.

### Clinical Site

The study will be conducted in the Third Cardiology Department of the Aristotle University of Thessaloniki (AUTH) in the Hippokration General Hospital, Thessaloniki, Greece. The hospital is equipped with a dedicated laboratory for ECHO acquisition (ECHO laboratory).

### Participants

ECHO scans will be acquired from 120 participants (data collection participants) referred for transthoracic echocardiography as part of routine clinical practice in Hippokration Hospital. In total, 15 cardiologists will be involved in the study (serving as comparators in phase 1), divided into three groups according to the relevant level of experience: (1) the low experience (LE) group will include residents of cardiology with ECHO experience of fewer than 5 years, (2) the medium experience (ME) group will include cardiologists with ECHO experience of more than 5 and less than 10 years, and (3) the highly experienced (HE) group will include cardiologists with more than 10 years of experience or certification in echocardiography from the European Association of Cardiovascular Imaging (EACVI) or the National Cardiac Society.

A panel of 3 expert cardiologists (reference panel) with more than 10 years of experience and certification in echocardiography from the EACVI or the national cardiac society, not participating in the HE group, will provide the reference measurements and diagnoses.

### Recruitment

Data collection participants will be recruited in the Third Cardiology Department of the Hippokration General Hospital. In particular, participants referred for transthoracic ECHO as part of routine clinical practice, including all possible diagnoses except those constituting exclusion criteria, will be invited to participate in data collection. Cardiologists serving at the Third Cardiology Department of the Hippokration General Hospital will be invited to participate in the examination of acquired echocardiograms.

### Inclusion and Exclusion Criteria for ECHO Acquisition

Participants eligible for data collection must meet the following criteria: (1) female or male, (2) older than 18 years, and (3) participants must be in sinus rhythm during the examination.

Participants will be excluded from data collection and processing based on the following criteria: (1) presence of atrial fibrillation, (2) presence of sinus or other tachycardia (heart rate>100 bpm), (3) presence of pacing rhythm, (4) complex congenital heart disease, (5) myocardial hypertrophy, (6) need for a contrast agent to improve echocardiographic resolution, (7) bad imaging quality with 2 or more myocardial segments not viewed in each apical view, and (8) incapability of providing full consent.

### Duration

The study is expected to last for approximately 2 years. The study is estimated to complete the enrollment of participants for data collection and of cardiologists within 6 months from initiation. However, enrollment in both cases will remain open until the study goal is met. Each cardiologist is expected to complete the tasks of both study phases in a maximum of 18 months. The second phase will only begin if and when the tool is proven to be noninferior in (at least) LV-EF measurement accuracy compared to (at least) the LE and ME groups of cardiologists in the first phase.

### Data Collection

#### Enrollment of Data Collection Participants and ECHO Scan Acquisition

Participants referred to the Third Cardiology Department of the Hippokration General Hospital for cardiovascular examination including ECHO (see “Recruitment” section) and deemed eligible for data collection will be invited to participate in the study. Enrollment in the study and ECHO scan acquisition will be performed on the same day. Participants will first review the study information sheet and undergo informed consent. Once consented, participants will be assigned a coded identification number. Afterward, basic demographics, anthropometrics, and vital signs (age, gender, height, weight, and blood pressure), as well as relevant medical history (known cardiovascular conditions), will be recorded. ECHO acquisition will follow the standard clinical practice. All ECHO scans will be performed at the Hippokration Hospital ECHO laboratory, by 1 experienced cardiologist with EACVI certification in ECHO. Scans will be acquired using the GE Vivid S5 ultrasound system (GE Healthcare) or the Clarius PA HD ultrasound scanner (Clarius Mobile Health Corp). Three views (a 2-chamber, a 3-chamber, and a 4-chamber apical view) will be acquired per scanning procedure, with 3 cardiac cycles per view. Views will be reviewed for diagnostic quality, and if a view does not meet the criteria set, the acquisition will be repeated.

#### Enrollment of Cardiologists

Cardiologists serving at the Hippokration Hospital will be invited to participate in the study. Cardiologists will first review the study information sheet and undergo informed consent. Once consented, they will be assigned a coded identification number. Demographics (age and gender) and experience, including career stage and experience in ECHO, will be recorded. Each cardiologist will be assigned to 1 of the 3 experience groups (LE, ME, and HE) according to the self-reported experience in ECHO.

#### Reference Measurements and Diagnoses

Acquired scans will be independently examined by the highly experienced cardiologists of the reference panel, who will estimate LV-EF and LV-GLS and provide a binary diagnosis of LV function (normal or abnormal) for each case. Examination of scans by the reference panel will take place on a single, dedicated computer (also referred to as a workstation) in the ECHO laboratory of the Hippokration Hospital. Cardiologists will examine scans in batches on different days, depending on their availability.

LV-EF and LV-GLS will be estimated using the same methods and standards applied in routine clinical practice. A commercially available speckle-tracking software (EchoPAC version 202.0, GE Healthcare) will be used. LV-EF will be estimated using the biplane Simpson method [4] from 2 and 4-chamber apical views after cardiologist-adjusted tracing of the LV cavity. For LV-GLS, the 2-dimensional speckle-tracking method [7] will be used. For each captured view, the cardiac cycle with the best image quality will be first selected out of the 3 captured cycles. If the image quality is similar among all 3 cycles, the intermediate one will be selected. To reduce bias due to user interaction, the beginning and end of the cardiac cycle will be the time points that will be automatically defined by the software, usually based on automatic QRS detection on the echocardiogram. In the second step, the endocardial border will be traced, and a region of interest will be constructed. Manual adjustments will be made after visual inspection of the segmental tracking results throughout the cardiac cycle, to obtain the best possible tracing of all myocardial segments. The peak systolic strain value per view, as automatically provided by the software, will be noted. Finally, LV-GLS will be calculated as the average peak strain of the 3 apical views.

Regarding LV-EF and LV-GLS, the reference measurement for each scan will be the average of measurements estimated by the 3 cardiologists, provided that there is a <20% discrepancy between the measurements. Regarding LV function, the reference diagnosis will be the majority diagnosis among the 3 equally weighted diagnoses, that is, at least 2 out of the 3 cardiologists of the reference panel arrived at this diagnosis. For cases where there is a discrepancy of ≥20% between at least 2 of the 3 LV-EF and LV-GLS measurements provided by the reference panel, the 3 cardiologists will examine them and estimate the reference measurements together.

#### Phase 1: Processing of ECHO Scans for LV-EF and LV-GLS Measurement

Out of the 120 ECHO scans to be acquired, the first 60 scans will be used for assessing the noninferiority of the AI-based tool in measuring LV-EF and LV-GLS.

Processing of scans by the AI-based tool will take place on the dedicated computer of the ECHO laboratory. For each scan, the AI-based tool will output an LV-EF and an LV-GLS measurement, along with the time required for producing both measurements. All 3 values will be recorded.

Comparator cardiologists of the 3 experience groups will independently examine each scan and estimate the LV-EF and LV-GLS. LV-EF and LV-GLS will be estimated using the same software and semimanual methods as in the case of the reference panel. For each scan, the LV-EF and LV-GLS values will be recorded along with the time required for estimation. The time required for estimation is defined as the time (in minutes) that elapsed from the moment a scan is loaded by the software until both measurements are estimated.

Cardiologists will examine scans in batches on different days, during working hours, and depending on their availability. A schedule with time slots will be communicated to them based on which they will be able to indicate their availability for the examination of each batch. Examination of scans will take place at the dedicated workstation of the ECHO Lab. At the end of phase 1, all 60 scans are expected to be processed by each cardiologist.

#### Phase 2: Examination of Scans for LV Function Diagnosis

In phase 2, the second batch of 60 scans will be used for assessing the superiority of the combination of the cardiologist and the AI-based tool in terms of LV function diagnosis from ECHO scans. Cardiologists of the 3 experience groups will independently examine batches of ECHO scans with and without the AI-based tool, and for each case, they will be asked to provide a binary diagnosis of LV function (normal or abnormal) based on visual inspection of the echocardiogram and measurements of LV-EF and LV-GLS. The decision will be recorded along with the time required to arrive at the diagnosis. For scans examined with AI assistance, cardiologists will use the AI-based tool that will allow them to view scans and automatically produce LV-EF and LV-GLS measurements. For scans examined without AI assistance, cardiologists will use the same software for viewing and estimating LV-EF and LV-GLS as in phase 1. For each cardiologist, ECHO scans reviewed with AI assistance will be different from those reviewed without AI assistance.

Before examining phase 2 cases, each cardiologist will be presented with materials describing how to use the AI-based tool and they will be given the opportunity to practice using the tool with 2 sample cases (independent of the study cases).

Examination of scans will take place on the same workstation of the ECHO laboratory as in phase 1, via scheduled time slots. At the end of phase 2, each cardiologist is expected to review and provide a diagnosis for 30 scans with AI assistance and 30 without AI assistance. Cardiologists that will examine at least 10 scans with AI assistance will be asked to complete the Greek version of the system usability scale (SUS).

### Outcomes

#### Phase 1

##### Primary Outcomes

The primary outcomes of phase 1 are the accuracy of LV-EF and LV-GLS measurements with respect to the reference measurements defined as the absolute error of estimated average LV-EF and the absolute error of estimated average LV-GLS over 3 apical views (2-chamber, 3-chamber, and 4-chamber), respectively.

##### Secondary Outcomes

The following secondary outcomes will be produced in phase 1: (1) a Bland-Altman plot of LV-EF measurements produced by the AI-based tool with respect to reference measurements; (2) Bland-Altman plots of LV-EF measurements produced by LE, ME, and HE cardiologists with respect to reference measurements; (3) a Bland-Altman plot of LV-GLS measurements produced by the AI-based tool with respect to reference measurements; (4) Bland-Altman plots of LV-GLS measurements produced by LE, ME, and HE cardiologists with respect to reference measurements; (5) intraclass correlation coefficient (ICC) of LV-EF measurements produced by the AI-based tool with reference measurements; (6) ICCs of LV-EF measurements produced by LE, ME, and HE cardiologists with reference measurements; (7) ICC of LV-GLS measurements produced by the AI-based tool with reference measurements; (8) ICCs of LV-GLS measurements produced by LE, ME, and HE cardiologists with reference measurements; (9) time required by the AI-based tool for producing LV-EF and LV-GLS measurements from an ECHO scan; and (10) time required by LE, ME, and HE cardiologists for producing LV-EF and LV-GLS measurements from an ECHO scan.

#### Phase 2

##### Primary Outcomes

The primary outcome of phase 2 is the agreement rate of the LV function diagnosis of LE, ME, and HE cardiologists with the reference diagnosis.

##### Secondary Outcomes

The secondary outcomes of phase 2 are the time required by LE, ME, and HE cardiologists for examining an ECHO scan and arriving at the diagnosis of LV function and the SUS score.

### Statistical Analysis Plan

#### Sample Size

The sample size of this study was determined based on realistic recruitment goals and comparability to the sample sizes of similar landmark studies [12,13]. In total, the study will recruit 120 individuals and each one will undergo a single ECHO examination. Half the scans will be used for phase 1 and the rest for phase 2. In total, 15 cardiologists, approximately 5 per experience group (LE, ME, and HE), will be recruited and participate in both study phases.

#### Statistical Analyses

In phase 1, errors of LV-EF and LV-GLS measurements produced by the AI-based tool will be compared to errors in the same measurements estimated semimanually by cardiologists, accounting for their experience level. We will use noninferiority tests for these comparisons. We will also compare the time required by the AI-based tool and by the comparator cardiologists to produce the measurements, using a superiority test. Bland-Altman analysis will also be performed to evaluate the agreement between the AI-based measurements, measurements by cardiologists of the 3 experience groups, and reference measurements and to detect bias and outliers. Moreover, ICC to assess interobserver variability regarding both LV-EF and LV-GLS measurements will be used. In phase 2, agreement rates of cardiologists’ diagnosis with the reference diagnosis with and those without the help of the AI-based tool will be compared. The same applies to the time to diagnosis. We will use superiority tests for these comparisons. Regarding usability, the SUS score will be interpreted according to a grade scale [14] and percentile ranking [15].

### Ethical Considerations

#### Approval

The study has been approved by the research ethics committee of AUTH, Thessaloniki, Greece (protocol 102808/2022). Any modifications to the protocol, which may impact the conduct of the study or may affect participant safety, will require a formal amendment to the protocol and subsequent review and approval by the research ethics committee of AUTH before the changes are implemented to the study.

#### Consent

Prospective participants will be provided with a participant information sheet and a consent form. They will be given sufficient time to read the material and ask any questions. Participants will be informed of the study aim, requirements for participation, risks and benefits, their rights, the usage and storage of personal data, and the contact for any issues related to the study. They will be explicitly informed that study participation is voluntary and that choosing against participation will not affect the care received for treatment, in the case of data collection participants, and their residency or work, in the case of cardiologists. Written and informed consent will be obtained from all participants before any study-related procedures.

#### Data Confidentiality and Management

All data collected will be handled in compliance with the General Data Protection Regulation (GDPR) of the European Union. The collection of personal participant information is limited to the amount necessary to achieve the aims of the research. Participants will be assigned a coded identification number that will be used for reference in all records to protect participant confidentiality through pseudonymization. All records, electronic or hard copy, will be securely stored in password-protected computers or locked cabinets, respectively, while those containing direct identifiers, such as signed consent forms, will be stored separately from other records, with limited access.

Only pseudonymized data will be used for data analysis. Investigators who are authorized by the principal investigator will be given access to the acquired, pseudonymized data sets. In case of data transfer over a computer network, this will take place via the secure shell (SSH) file transfer protocol (SFTP). Study participants will be able to request access, correction, limited processing, or deletion of the data they contributed to the study, in compliance with the respective mandates of the GDPR. Participants will also have the right to data portability.

#### Withdrawal

All participants, including data collection participants and cardiologists, are free to withdraw from participation at any time, for any reason, specified or unspecified, and without prejudice. Participants can also request for the data they contributed to be deleted. Reasonable attempts will be made by the investigator to provide a reason for participant withdrawals.

#### Safety

Data will be collected from participants referred for echocardiography based on routine clinical practice. Thus, there are no additional safety concerns arising from their participation in the study. Standard clinical procedures will be followed for acquiring ECHO scans. Any distress or other adverse effects that may occur during ECHO will be handled according to standard clinical practice. Measurements and diagnoses produced during both phases of the study will only be used for evaluating the performance and utility of the AI-based tool, and they will not affect the formal diagnosis, treatment, or care that the participant will receive, which will be based on standard clinical practice.

## Results

Enrollment began in September 2022. Participant recruitment and ECHO scan collection are ongoing. The results of the first phase are expected by summer 2023. The study is expected to conclude in May 2024, with the completion of the second phase.

## Discussion

### Principal Anticipated Findings

This is a 2-phase, prospective cohort study aiming at externally validating the clinical performance of and producing preliminary utility evidence regarding an AI-based tool that automatically estimates LV-EF and LV-GLS from transthoracic ECHO scans. ECHO scans will be collected from 120 participants referred for ECHO examination in the Hippokration General Hospital, Thessaloniki, Greece. The first phase will evaluate the accuracy of the AI-based tool, its reliability, and measurement time compared to semimanual estimations of cardiologists of different experience levels. We expect to find that the AI-based tool is noninferior in terms of LV-EF and LV-GLS estimation accuracy compared to at least LE and ME cardiologists, yields more consistent measurements, and is superior in terms of time required for LV-EF and LV-GLS estimation compared to cardiologists of all experience levels.

Provided that the AI-based tool proves noninferior in terms of at least LV-EF estimation accuracy compared to at least LE and ME cardiologists, the second phase will determine whether the tool can assist cardiologists of different experience levels in classifying LV function as normal or abnormal correctly and in less time. We expect to find that the combination of the cardiologist and the AI-based tool is superior in terms of agreement with the reference LV function diagnosis (normal or abnormal) and time to diagnosis compared to the cardiologist’s routine examination practice, at least in the cases of LE and ME cardiologists. The second phase will also yield usability evidence regarding the tool.

### Comparison to Prior Work

To our knowledge, at the time of approval, this was the first prospective cohort study aiming to evaluate the clinical performance and utility of an AI-based tool for automatic estimation of both LV-EF and LV-GLS from ECHO scans. Prior studies focused on evaluating the performance of AI-based tools for either EF or GLS automatic estimation. In the study of Salte et al [13], the performance of an AI-based system for automatic LV-GLS estimation was assessed by comparing AI-based GLS measurements to reference, semiautomatic GLS measurements by a single experienced observer in 200 ECHO scans. Pearson correlation coefficient, the mean absolute difference, and Bland-Altman analysis metrics were used to compare the 2 types of measurements, with LV function (measured using LV-EF) and the quality of scans considered as covariates. The study also investigated intraobserver variability by comparing initial GLS measurements with measurements performed after 4 weeks by the same observer in 25 ECHO scans, as well as interobserver variability by comparing the GLS measurements of the experienced observer to measurements by a second experienced observer performed on the same 25 scans. In a recent work [16], a retrospective study was carried out to compare LV-EF measurements of an AI-based tool to physician-measured LV-EFs with cardiac magnetic resonance imaging–derived EFs as the reference. ECHO scans from 242 patients were used, while physician-measured LV-EFs were obtained from formal clinical reports. Pearson correlation coefficient and Bland-Altman analysis metrics were used for comparisons, with the use or not of ultrasound-enhancing agent during ECHO acquisition considered as a covariate.

### Strengths and Limitations

This study has several strengths. First, it will generate external evidence regarding the clinical performance of a trained AI-based tool for automatic estimation of LV-EF and LV-GLS based on prospective data collected in the routine clinical setting, thus reflecting real-world clinical scenarios. Second, it will provide preliminary evidence of the utility of such a tool in the diagnosis of LV function. Last, it will take into account the experience of the cardiologist in ECHO as a covariate both in EF and GLS measurement, as well as the diagnosis of LV function. A key limitation of this study is that the sample size was not determined through exhaustive power analysis but based on a realistic recruitment rate and sample sizes of previous studies with similar objectives and outcomes.

### Dissemination Plan

After the completion of the study, we plan to publish the findings in peer-reviewed, open-access biomedical journals with a multidisciplinary readership spanning medicine, data science, and engineering. We will also report on the findings in deliverables and on the website of the associated project, at medical and biomedical engineering conferences, as well as in events with relevant stakeholders. The publication or presentation of any study findings will be done with respect to participants’ privacy. Publications or presentations will not include any identifying participant information.

### Future Directions

Depending on the findings of the study, future work may include the design and conduct of a randomized control trial to comprehensively evaluate the clinical utility of the AI-based tool, that is, its potential to lead to improved patient outcomes when used in a specific clinical setting.

### Conclusions

Through the present study, we anticipate generating evidence on the clinical performance and utility of an AI-based tool for the automatic estimation of LV-EF and LV-GLS from echocardiograms. Such a tool has the potential to advance the process of ECHO examination through accurate, consistent, and fast estimation of key LV functional parameters that may lead to improved patient outcomes, especially in demanding care settings such as emergency rooms. Validation of clinical performance and preliminary proof of utility are the first steps in the assessment process of the tool toward its regulatory approval and use in clinical practice. The detailed description of the protocol presented here may be useful for clinicians and researchers planning similar studies.

## Appendix

Multimedia Appendix 1 Peer review report by European Union Horizon 2020 - Research and Innovation Framework Programme.

## Abbreviations

AI: artificial intelligence
AUTH: Aristotle University of Thessaloniki
EACVI: European Association of Cardiovascular Imaging
ECHO: echocardiography
EF: ejection fraction
GDPR: General Data Protection Regulation
GLS: global longitudinal strain
HE: high experience
ICC: intraclass correlation coefficient
LE: low experience
LV: left ventricle or left ventricular
ME: medium experience
SFTP: secure shell file transfer protocol
SSH: secure shell
SUS: system usability scale

## Data Availability

The data sets generated and analyzed during the current study are not publicly available due to GDPR compliance and in order to safeguard participants’ privacy but will be available from the corresponding author on reasonable request.
