# Supplementary material for: Clinical Validation of an Artificial Intelligence–Based Tool for Automatic Estimation of Left Ventricular Ejection Fraction and Strain in Echocardiography: Protocol for a Two-Phase Prospective Cohort Study
Source: JMIR Res Protoc. 2023 Mar 13;12:e44650. doi: 10.2196/44650 (PMC10131996; doi:10.2196/44650)
Supplement: Multimedia Appendix 1 [file resprot_v12i1e44650_app1.pdf]

Proposal Evaluation Form

EUROPEAN COMMISSION

Horizon 2020 - Research and Innovation Framework Programme

Evaluation Summary Report - Innovation actions

**Call:**	H2020-DT-2020-1
**Type of action:**	IA
**Proposal number:**	101016834
**Proposal acronym:**	HosmartAI
**Duration (months):**	40
**Proposal title:**	Hospital Smart development based on AI
**Activity:**	DT-ICT-12-2020 IA

N.	Proposer name	Country	Total Cost	%	Grant Requested	%
1	INTRASOFT INTERNATIONAL	BE	1,036,625	8.71%	725,637	7.26%
2	PHILIPS MEDICAL SYSTEMS NEDERLAND BV	NL	1,075,750	9.04%	753,025	7.53%
3	VIMAR SPA	IT	732,626.25	6.16%	512,838	5.13%
4	GREEN COMMUNICATIONS SAS	FR	427,500	3.59%	299,250	2.99%
5	TELEMATIC MEDICAL APPLICATIONS EMPORIA KAI ANAPTIXI PROIONTON TILIATRIKIS MONOPROSOPIKI ETAIRIA PERIORISMENIS EYTHINIS	EL	410,250	3.45%	287,175	2.87%
6	ECLEXYS SAGL	CH	741,160	6.23%	518,812	5.19%
7	F6S NETWORK LIMITED	IE	271,625	2.28%	190,137	1.90%
8	Pharmecons Easy Access Ltd	UK	300,250	2.52%	210,175	2.10%
9	TERAGLOBUS LATVIA SIA	LV	278,375	2.34%	194,862	1.95%
10	Ninety One GmbH	DE	360,250	3.03%	252,175	2.52%
11	EIT Health Germany GmbH	DE	368,500	3.10%	257,950	2.58%
12	Univerzitetni klinicni center Maribor	SI	306,500	2.58%	306,500	3.07%
13	SAN CAMILLO IRCCS S.R.L.	IT	335,250	2.82%	234,675	2.35%
14	SERVICIO MADRILENO DE SALUD	ES	198,375	1.67%	198,375	1.98%
15	CENTRE HOSPITALIER UNIVERSITAIRE DE LIEGE	BE	361,500	3.04%	361,500	3.62%
16	UNIVERSITY GENERAL HOSPITAL OF THESSALONIKI AHEPA	EL	225,000	1.89%	225,000	2.25%
17	VRIJE UNIVERSITEIT BRUSSEL	BE	589,500	4.95%	589,500	5.90%
18	ARISTOTELIO PANEPISTIMIO THESSALONIKIS	EL	517,750	4.35%	517,750	5.18%
19	EIDGENOESSISCHE TECHNISCHE HOCHSCHULE ZUERICH	CH	545,913.75	4.59%	545,913	5.46%
20	UNIVERZA V MARIBORU	SI	442,750	3.72%	442,750	4.43%
21	INSTITUTO TECNOLÓGICO DE CASTILLA Y LEON	ES	1,362,125	11.45%	1,362,125	13.63%
22	FUNDACION INTRAS	ES	548,100	4.61%	548,100	5.48%
23	ASSOCIATION EUROPEAN FEDERATION FORMEDICAL INFORMATICS	CH	255,000	2.14%	255,000	2.55%
24	FEDERATION EUROPEENNE DES HOPITAUX ET DES SOINS DE SANTE	BE	208,020	1.75%	208,020	2.08%
	Total:		11,898,695		9,997,244	

Abstract:
HosmartAI will create a common open Integration Platform with the necessary tools to facilitate and measure the benefits of integrating digital technologies (robotics and AI) in the healthcare system. A central hub will offer multi-faceted lasting functionalities (Marketplace, Co-creation space, Benchmarking) to healthcare stakeholders, combined with a collection of methods, tools and solutions to integrate and deploy AI-enabled solutions. The Benchmarking tool will promote the adoption in new settings, while enabling a meeting place for technology providers and end-users. Eight Large-Scale Pilots will implement and evaluate improvements in medical diagnosis, surgical interventions, prevention and treatment of diseases, and support for rehabilitation and long-term care in several Hospital and care settings. The project will target different medical aspects or manifestations such as Cancer (Pilot #1, #2 and #8); Gastrointestinal (GI) disorders (Pilot #1); Cardiovascular diseases (Pilot #1, #4, #5 and #7); Thoracic Disorders (Pilot #5); Neurological diseases (Pilot #3); Elderly Care and Neuropsychological Rehabilitation (Pilot #6); Fetal Growth Restriction (FGR) and Prematurity (Pilot #1). To ensure a user-centred approach, harmonization in the process (e.g. regarding ethical aspects, standardization, and robustness both from a technical and social and healthcare perspective), the living lab methodology will be employed. HosmartAI will identify the appropriate instruments (KPI) that measure efficiency without undermining access or quality of care. Liaison and co-operation activities with relevant stakeholders and open calls will enable ecosystem building and industrial clustering. HosmartAI brings together a consortium of leading organizations (3 large enterprises, 8 SMEs, 5 hospitals, 4 universities, 2 research centres and 2 associations) along with several more committed organizations (Letters of Support provided).

Evaluation Summary Report

Evaluation Result

Total score: 14.00 (Threshold: 10)

Form information

SCORING

Scores must be in the range 0-5.

Interpretation of the score:

__0__ The __proposal fails to address the criterion__ or cannot be assessed due to missing or incomplete information.

__1 Poor.__ The criterion is inadequately addressed, or there are serious inherent weaknesses.

__2 Fair.__ The proposal broadly addresses the criterion, but there are significant weaknesses.

__3 Good.__ The proposal addresses the criterion well, but a number of shortcomings are present.

__4 Very good.__ The proposal addresses the criterion very well, but a small number of shortcomings are present.

__5 Excellent.__ The proposal successfully addresses all relevant aspects of the criterion.Any shortcomings are minor.

Criterion 1 - Excellence

Score: **4.50** (Threshold: 3/5.00 , Weight: -)

The following aspects will be taken into account, to the extent that the proposed work corresponds to the topic description in the work programme:
Clarity and pertinence of the objectives
Soundness of the concept, and credibility of the proposed methodology
Extent that proposed work is beyond the state of the art, and demonstrates innovation potential (e.g. ground-breaking objectives, novel concepts and approaches, new products, services or business and organisational models)
Appropriate consideration of interdisciplinary approaches and, where relevant, use of stakeholder knowledge and gender dimension in research and innovation content

HosmartAI addresses three priorities: secure access to personal health data; personalized medicine through shared pan European data infrastructure; empowering citizens with digital tools for user-centred care.
The objectives are focused on integrating robotics and AI in an open Platform to be applied in 8 extensive pilots.
Addressing a broad range of health conditions and treatments, the proposal is in the scope of the call. There is evidence of close connection with Digital Innovation Hub DIH-Hero and AI4EU platform.
The objectives are clearly established and defined, pertinent, achievable and innovative. The objectives are balanced to address business, technology and innovation aspects; each has a list of means and measures (KPIs) to achieve outcomes and assure assessment.
The concept is sound and clear and has a solid background. Multiple medical aspects will be addressed.
Overall the methodology is credible. Pilots will include clinical as well as process outcomes and productivity measures before and after the introduction of the new systems, such as improvement in clinical practice, increased patient and clinical staff satisfaction rates, improved hospital productivity indicators in an efficient (cost-effective) way.
Some minor shortcomings are that the proposal does not sufficiently discuss a method to support personnel in prioritizing training for this new technology; networking and cloud technologies and architectures are not fully described.
Trust and acceptance are described. However, how they are measured and how trust is built is not sufficiently described in the proposal.
The proposal has a very good innovation potential of the technology and it goes beyond the current state-of-the art in terms of technology demonstration.
KPIs are included and well described.
The end users and stakeholders are involved in the proposal through engagement and participatory design activities.
Interdisciplinarity is considered and partners bring in relevant business, technology and scientific expertise.
There is appropriate consideration of the gender dimension in research and innovation content.

Criterion 2 - Impact

Score: **5.00** (Threshold: 3/5.00 , Weight: -)

The following aspects will be taken into account:
The extent to which the outputs of the project would contribute to each of the expected impacts mentioned in the work programme under the relevant topic
Any substantial impacts not mentioned in the work programme, that would enhance innovation capacity, create new market opportunities, strengthen competitiveness and growth of companies, address issues related to climate change or the environment, or bring other important benefits for society
Quality of the proposed measures to:
- exploit and disseminate the project results (including management of IPR), and to manage research data where relevant
- communicate the project activities to different target audiences

The proposal is expected to contribute to each of the impacts mentioned in the work programme, at pan-European level. It is structured to reach genuine, novel and positive impact on health, ethical and socio-economical elements, on technological competitiveness, and on the establishment of a sustainable business ecosystem.
Existing barriers (technological, social, business, economic) will be examined throughout the duration of the proposed project in order to adapt to their impact on the innovation scopes.
The size of the planned pilots is very large (3000 patients, 300 healthcare professionals, 600 stakeholders/managers) in different contexts (care-homes, primary care settings, secondary and tertiary care or rehabilitation centers) in a series of pan European AI based pilots for the smart hospital of the future; hence, good scientific and technological impact is expected. New functionalities, open APIs and all platform components will be released as open source, thus enhancing the impact, directly or indirectly, on the overall community.
HosmartAI has been structured from the beginning to match the requirements for being a first seller within the reality of a European AI as an “Ecosystem of Trust” and “Ecosystem of Excellence”, which EU institutions are already promoting.
Among the described KPIs for evaluation of impact, one KPI deals with trust and acceptance of the system.
The proposal also has potential on other aspects such as societal, including the digital single market industry for Europe.
Environmental footprint, training and safety of healthcare workforce are considered, which is commendable.
The proposal uses technology developed through other sources of funding, hence leverages the outcomes of previous projects.

Appropriate measures to maximize impact have been described and justified. The dissemination strategy is clear and well justified; it includes academia, business and healthcare community addressing general public and decision makers too.
Exploitation plans are very well developed and accounted for in the proposal. They are described and separated into a few phases during the project life and after. A preliminary business plan is provided, which is appropriate.
Standards, privacy, security, ethics are considered; the project outcomes will be accessible through OpenAPIs.
Research and Data Management are described and further developed in a dedicated WP. IPR will be adequately managed.
A communication plan is described, targeting all relevant communities and target audiences, including industry, governmental decision makers and policy formulators, investors, end-users.

Criterion 3 - Quality and efficiency of the implementation

Score: **4.50** (Threshold: 3/5.00 , Weight: -)

The following aspects will be taken into account:
Quality and effectiveness of the work plan, including extent to which the resources assigned to work packages are in line with their objectives and deliverables
Appropriateness of the management structures and procedures, including risk and innovation management
Complementarity of the participants and extent to which the consortium as a whole brings together the necessary expertise
Appropriateness of the allocation of tasks, ensuring that all participants have a valid role and adequate resources in the project to fulfil that role

The work plan is clear even if complex (the project has 24 partners), as many different partners of different nature will be involved in the proposal. However, the methodology to tackle the potential coordination issue due to the involvement of a large number of participants is not sufficiently discussed.
The tasks in the WPs are logically and well described, and they map to the objectives and deliverables.
The work plan is strengthened by providing financial support to third parties. It is not clear whether the pilots will materialize into applications after the end of the proposed project.
The management structure and procedures are well described and appropriate for the size of the proposal and its multi-stakeholder, multi-location nature. A project management and quality handbook will be produced with detailed criteria and rules and this adds efficiency of management and quality. Task 9.3 will ensure that the scientific and technical results of the proposal will be produced on the basis of high-quality standards. The decision-making, voting, and conflict resolution management has also been very well described in the proposal. The formation of an external Advisory Board is an asset.
Administrative, business risks and contingency plans are very well described, with a detailed list provided. The technical and scientific risks are general and, as a result, mitigation measures are not sufficiently described.
The Innovation management is appropriately described.
The partners bring the necessary expertise and as a whole are very complementary and will be able to carry out all the tasks described in the proposal. The roles are matched with the proposed objectives.
Resources are appropriately distributed among WPs according to the work, and across partners according to their role.

Scope of the proposal

Status: **Yes**

Comments (in case the proposal is out of scope)

Not provided

Operational Capacity

Status: **Operational Capacity: Yes**

If No, please list the concerned partner(s), the reasons for the rejection, and the requested amount.

Not provided

Exceptional funding of third country participants/international organisations

A third country participant/international organisation not listed in General Annex A to the Main Work Programme may exceptionally receive funding if their participation is essential for carrying out the project (for instance due to outstanding expertise, access to unique know-how, access to research infrastructure, access to particular geographical environments, possibility to involve key partners in emerging markets, access to data, etc.). (For more information, see the Online Manual)

Based on the information provided in the proposal, we consider that the following participant(s)/international organisation(s) that requested funding should exceptionally be funded:
(Please list the Name and acronym of the applicant, Reasons for exceptional funding and the Requested grant amount.)

Not provided

Based on the information provided in the proposal, we consider that the following participant(s)/international organisation(s) that requested funding should NOT be funded:
(Please list the Name and acronym of the applicant, Reasons for exceptional funding and the Requested grant amount.)

Not provided

Use of human embryonic stem cells (hESC)

Status: **No**

If yes, please state whether the use of hESC is, or is not, in your opinion, necessary to achieve the scientific objectives of the proposal and the reasons why. Alternatively, please state if it cannot be assessed whether the use of hESC is necessary or not because of a lack of information.

Not provided

Overall comments

Not provided

FUNDING & TENDERS PORTAL E-RECEIPT

Digitally sealed by the European Commission
Date: 2020.09.21 11:11:06 CEST

This electronic receipt is a digitally signed version of the document submitted by your organisation. Both the content of the document and a set of metadata have been digitally sealed.

This digital signature mechanism, using a public-private key pair mechanism, uniquely binds this eReceipt to the modules of the Funding & Tenders Portal of the European Commission, to the transaction for which it was generated and ensures its full integrity. Therefore a complete digitally signed trail of the transaction is available both for your organisation and for the issuer of the eReceipt.

Any attempt to modify the content will lead to a break of the integrity of the electronic signature, which can be verified at any time by clicking on the eReceipt validation symbol.

More info about eReceipts can be found in the FAQ page of the Funding & Tenders Portal.

(https://ec.europa.eu/info/funding-tenders/opportunities/portal/screen/support/faq)
